# Psychotropic Medication of Acute Episodes of Mood Disorders: Current Prescription Attitude in Two Psychiatric Wards in Cagliari, Italy

**DOI:** 10.2174/1745017901814010236

**Published:** 2018-10-30

**Authors:** Gioia Baggiani, Luca Ambrosiani, Pierfranco Trincas, Caterina Burrai, Alberto Bocchetta

**Affiliations:** 1 Section of Neuroscience and Clinical Pharmacology, Department of Biomedical Sciences, University of Cagliari, Cagliari, Italy; 2 Psychiatric Ward Unit 2, “Santissima Trinità Hospital”, ATS Sardegna, ASSL Cagliari, Cagliari, Italy; 3 Psychiatric Ward Unit 1, “Santissima Trinità Hospital”, ATS Sardegna, ASSL Cagliari, Cagliari, Italy

**Keywords:** Mood disorders, Bipolar and related disorders, Suicide, Lithium, Valproic Acid, Carbamazepine, Lamotrigine, Antipsychotic agents

## Abstract

**Background:**

Medication of acute episodes of mood disorders has changed over the last decades following the results of randomized clinical trials.

**Objective:**

The aim of this study was to analyze medication prescribed at discharge from two psychiatric wards. We focused on hospitalization as one of the best opportunities to start prophylaxis.

**Methods:**

We examined retrospectively the clinical records of 357 patients hospitalized for mood episodes in two psychiatric wards in the Cagliari area (SPDC-1 and SPDC-2) between 1 January and 31 December 2016. We focused on the psychotropic medication prescribed at discharge from the hospital.

**Results:**

Most patients were discharged with antipsychotics (86%) and/or benzodiazepines (89%). Combined medication was frequent, including various co-administration of first-generation and/or second-generation antipsychotics (26% of patients), or antipsychotics combined with mood-stabilizers (51% of patients). There was a preferential prescription of first-generation antipsychotics in SPDC-1, and of second-generation antipsychotics in SPDC-2. Prescription of lithium was significantly more frequent in SPDC-1.

**Conclusion:**

Although the treatment was in line with randomized clinical trials, the choice of individual psychotropic agents differed significantly between the two wards. Different prescription attitudes can have consequences on the long-term outcome of patients discharged from the hospital after an acute mood episode.

## 1 INTRODUCTION

Psychotropic medication of acute episodes of mood (affective) disorders has changed over the last decades. In particular, second-generation antipsychotics and anticonvulsants have progressively substituted first-generation antipsychotics and lithium in the treatment of acute episodes of bipolar disorder [1]. A series of randomized clinical trials has resulted in the approval of several drugs by regulatory agencies [2,3]. However, the long-term outcome after an acute episode has rarely been the subject of controlled studies [4]. This represents an unmet need because mood disorders tend to recur, are associated with a high risk of suicide, and prophylaxis is crucial [5]. In the case of recurrences requiring hospitalization, the prescription attitude of healthcare professionals has changed a lot over time. In particular, the prescription of lithium therapy varied through the years despite its established efficacy in the long-term outcome [6] and its unique suicide-preventing property [7].

As reviewed by Perugi and Fornaro [8], several national and international guidelines have been developed to provide a synthesis of current scientific knowledge developed by the integration of the evidence-based data with the rational clinical practice and the experience. However, there may be a divergence between common clinical practice attitudes and the existing guidelines. Guidelines warrant continuous updates: An example is provided in the UK by the National Institute for Health and Care Excellence (NICE) [9].

The debate is not merely academic because prescription choices can influence public health and have a relevant socioeconomic impact.

Our group has been involved in research and clinical work of mood disorder over the last four decades. In particular, CB and AB started their professional career in the late 1970s, when lithium therapy was introduced in Sardinia, and have experienced the changes in both diagnostic instruments and medication armamentarium. We have noticed that prescription attitudes have apparently resulted in a decline in the use of lithium in Cagliari area, despite the growing literature evidence of its superiority in specific circumstances. The lack of initiatives from local institutional agencies to explore this phenomenon prompted us to undertake this study, with a secondary aim of contributing to the global field beyond the local region.

The principal aim of this study was to analyze the psychotropic medication prescribed at discharge from two psychiatric wards to the patients hospitalized for mood (affective) episodes over one calendar year. We focused particularly on lithium therapy, based on the consideration that a hospital stay is one of the best opportunities to start (or reinforce) prophylaxis.

## 2 MATERIALS AND METHODS

We examined retrospectively the clinical records of the patients hospitalized for affective episodes (ICD-10 F30-F39) in two psychiatric wards in the Cagliari area between January 1 and December 31, 2016. According to the reform introduced in Italy in 1978, SPDC (Servizio Psichiatrico di Diagnosi e Cura = Psychiatric Diagnosis and Care Service) provides for the needs of patients requiring medical treatment involving a stay in the hospital, both in the case of voluntary admissions and in that of compulsory treatment [10]. In the Cagliari area, up to December 2008, there was a single SPDC with 30 hospital beds for the approximately 300,000 inhabitants. Thereafter, the service was divided into two wards (for which we will use the names SPDC-1 and SPDC-2). Both services are part of the framework of the general hospital “Santissima Trinità” with 15 and 12 hospital bed, respectively. For the purpose of this study, we extracted the following data from the charts of patients who had been hospitalized at least once in 2016 for an affective episode: Demographic characteristics, diagnosis, age of onset of the disorder, prior psychotropic medication with particular attention to lithium treatment, number of prior hospitalizations, history of suicide attempts and history of alcohol or drug abuse. We also focused on the psychotropic medication prescribed at discharge from the hospital. In 28 cases, patients were hospitalized more than once during 2016, therefore the last hospitalization was chosen as the index one.

## 3 RESULTS

Demographic and clinical characteristics of the 357 patients studied are shown in Table **1**. The duration of stay in the hospital ranged between one day and approximately six weeks. The mean duration was longer for SPDC-1.

The two wards differed in the proportion of ICD-10 diagnoses Fig. (**1**). In particular, SPDC-1 patients were diagnosed more often with “classical” bipolar disorder (F30-F31), whereas SPDC-2 patients were more often diagnosed with unspecified (F39) or schizoaffective (F25) disorders.

The great majority of patients were discharged from the two psychiatric wards with antipsychotics (306/357 = 86%) and benzodiazepines (317/357 = 89%). At discharge, apart from benzodiazepines, there was an extensive prescription of combined medication, including 92/357 (26%) cases with various combinations of first-generation and/or second-generation antipsychotics, and 181/357 (51%) cases with antipsychotics combined with mood-stabilizers.

**Fig. (1):**
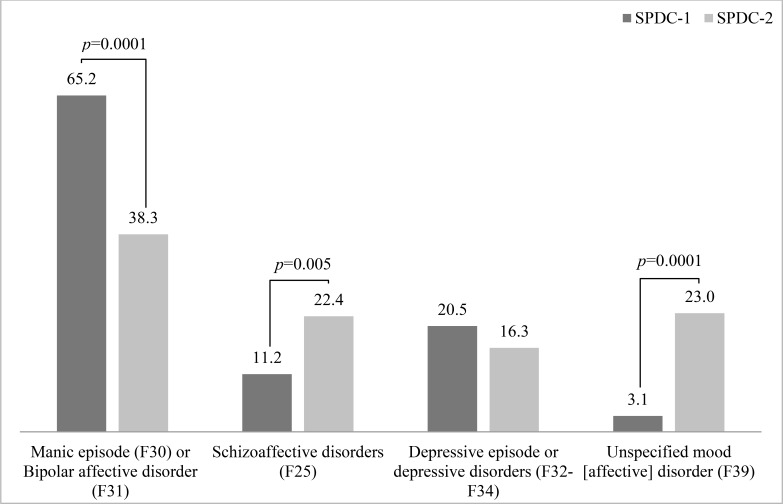
Rates (%) of ICD-10 diagnoses in patients hospitalized in the two psychiatric wards SPDC-1 (n = 161) and SPDC-2 (n = 196).

With regard to specific classes of drugs, there was a preferential prescription of first-generation antipsychotics in SPDC-1 and a preferential prescription of second-generation antipsychotics in SPDC-2 Fig. (**2**). A slightly non-significant higher proportion of patients from SPDC-1 was discharged with antidepressants, probably reflecting the slight (non-significant) greater proportion of depressive episodes in SPDC-1 (Fig. **1**).

Principal individual drugs prescribed at discharge from the two wards are shown in Table **2**. Haloperidol was the most prescribed first-generation antipsychotic in both wards, followed by promazine. Quetiapine was the most prescribed second-generation antipsychotic in both wards, followed by aripiprazole.

In any case, haloperidol was prescribed more often in SPDC-1 than SPDC-2, whereas the two-second generation antipsychotics olanzapine and aripiprazole were prescribed more often in SPDC-2 than SPDC-1.

Prescription at the discharge of anticonvulsants officially approved for mood disorders (carbamazepine, valproate, and lamotrigine) was similar between the two SPDCs, either as a class Fig. (**2**) or as individual compounds Table **2**. In 25 cases, treatment at discharge included anticonvulsants that are not officially approved in Italy for mood disorders (gabapentin, topiramate, oxcarbazepine, and pregabalin).

The attitude of prescribing lithium at discharge differed significantly between the two SPDCs Fig. (**2**) and Table **2**.

In particular, of the 53 patients who had already been treated with lithium before the index episode, a greater proportion of those hospitalized in SPDC-1 was discharged again with lithium compared to those in SPDC-2 (15/24 = 62% *versus* 4/29 = 14%; *p* = 0.0004). Of the 102 patients with a history of prior suicide attempts, lithium was prescribed at discharge to a greater proportion of patients from SPDC-1 than SPDC-2 (19/52 = 37% versus 7/50 = 14%; *p* = 0.0121).

## 4 DISCUSSION

This survey of acute mood (affective) episodes requiring hospitalization in two psychiatric wards was undertaken to test the current prescription attitude in the “real world”, compared to the context of treatment guidelines and randomized clinical trials. The latter has resulted in the approval of several new drugs (principally antimanic agents) by regulatory agencies from different countries. Prescription patterns have changed over the last two decades in favor of newer medications. This shift, however, may not be completely based on evidence with regard to the therapeutic phases that follow an acute episode (*eg*. continuation, maintenance, prophylaxis). These results confirm that, even if regulatory recommendations are followed with regard to acute treatments, there may be wide variations in treatment choices, that may have consequences in terms of public health and have a relevant socioeconomic impact (such as subsequent recurrence rate, quality of life, tolerability, suicide prevention, *etc*).

### 4.1 Old Antimanic Agents (Lithium and First-Generation Antipsychotics)

The first modern medication in mood disorders was introduced almost 70 years ago but still represents the gold standard. In fact, the Australian psychiatrist John Cade described the antimanic properties of lithium in 1949 [11]. Five years later, Mogens Schou and his colleagues implemented the first placebo-controlled double-blind trial in psychopharmacology, confirming the antimanic properties of lithium [12]. Subsequent controlled studies in the early 1970s provided evidence that lithium is also able to prevent recurrences of bipolar disorder [13]. Thereafter, there has been a great evolution in the design of clinical trials across the various phases of bipolar disorder. The efficacy of treatments for acute mania has been established, replicated and accepted widely [14]. The armamentarium has extended, as witnessed by the great variety of compounds prescribed to the patients from this study. As controlled trials often conclude for non-inferiority of a new active compound versus a comparison active medication, the therapeutic choice is left to the preferences of the individual clinicians.

With regard to acute mania, many drugs have been approved in different countries since the 1970s. For example, the US Food and Drug Administration (FDA) approved lithium in 1970 and the neuroleptic chlorpromazine in 1973. Other medications have long been used in acute mania but not necessarily approved by regulatory authorities. Paradoxically, haloperidol, which is not FDA-approved has been used as a comparison treatment in many modern trials of acute mania, as reviewed by Cipriani and colleagues [15]. Haloperidol is not promoted any more by the manufacturer due to its long stay in the market, nevertheless, it was one of the most prescribed individual medication to the patients from this study.

### 4.2 Clozapine

Another example of the discrepancies between the real world and randomized clinical trials is clozapine, which has been used as monotherapy or as add-on therapy in particular in the case of treatment-resistant mania, but whose effects were addressed by uncontrolled trials alone [16, 17].

Clozapine was prescribed to a small proportion of patients from this study (approximately 5%), supposedly those with treatment-resistant episodes. One reason for clozapine's limited use is its side-effect profile (including constipation, hypersalivation, weight gain), and its association with the serious adverse effect of agranulocytosis [18]. The latter is fairly uncommon and can be prevented in long-term maintenance by monthly blood-counts, mandatory in most countries, including Italy. However, it must be underscored that clozapine may have antisuicide properties, as shown at least in patients diagnosed with schizophrenia [19, 20]. According to a Swedish population-based cohort study, clozapine shows a positive benefit-risk ratio when comparing the benefit of reducing suicide and suicide attempts to the risk of agranulocytosis [21].

### 4.3 Anticonvulsants

Carbamazepine was prescribed to less than 10% of patients discharged from SPDC-1 and SPDC-2. Carbamazepine has been used in the treatment of various phases of bipolar disorder since the early 1980s [22] but was eventually approved by FDA in 2004 for acute mania alone. In this case, the development of an extended-release capsule formulation prompted the two randomized placebo-controlled trials required for approval [23, 24]. In Europe, the use of carbamazepine for mood disorders was launched in the 1980s but is now in decline for several reasons, including its relatively slow time to efficacy and its side-effect profile (dizziness, ataxia, diplopia, fatigue, and nausea). A slow titration is usually recommended in the treatment of acute episodes (much slower compared to valproate). The declining use of carbamazepine could also be attributed to a reduced interest by the manufacturer.

There have been some studies comparing carbamazepine to lithium in the maintenance phase of mood disorder, finding it somewhat less effective [25, 26]. In our experience, adding carbamazepine to lithium can restore prophylactic effectiveness in patients with bipolar disorder who proved refractory to lithium monotherapy [27]. In Italy, there is an example of the influence of “non-scientific” reasons for the changing attitude of prescribing anticonvulsants in mood disorders. Despite valproate’s antimanic properties had been reported since the 1960-1970s, Italian psychiatrists had to prescribe valproate “off-label” for many years or use the analog valpromide that had been approved earlier as “coadjuvant in depressive states and manic excitement, and in psychomotor agitation”. Eventually, after the introduction of a prolonged-release valproate formulation in Italy, the manufacturer applied for and obtained approval for treatment and prevention of mania associated with bipolar disorder in the 2000s. In the US, valproate semisodium had been approved in 1995 for acute mania and a dramatic change in prescription patterns followed at the expense of lithium [28-30].

The relatively small number of patients (3%) treated with lamotrigine at discharge from SPDC-1 and SPDC-2 can be attributed to the probable excess of patients with predominant manic than depressive episodes. Moreover, lamotrigine did not demonstrate efficacy in four out of five placebo-controlled clinical studies in the acute treatment of bipolar depression [31], as opposed to its prophylactic effect on depressive recurrences [32]. However, there has been a debate about this so-called “disconnect between initial judgments of lamotrigine *versus* its real-world effectiveness in managing bipolar disorder” [33, 34].

Focusing on the challenging problem of bipolar depression, Ketter [35] stated that “some FDA-approved treatments are helpful during acute and maintenance phases of therapy, but there is a significant unmet need for effective bipolar depression treatments with favorable side-effect profiles. Newer agents offer the promise of improvements in tolerability, but additional research is needed to actualize this promise into better treatments for patients struggling with bipolar depression” [35].

Other anticonvulsants, such as gabapentin, topiramate, oxcarbazepine, and pregabalin, were also prescribed to some patients included in this survey, even if their efficacy has not been established by controlled studies [36-38].

### 4.4 Second-Generation Antipsychotics

Between 2000 and 2004, there has been an “explosion” of controlled trials that resulted in FDA approval for acute mania of the second-generation antipsychotics olanzapine, risperidone, quetiapine, ziprasidone and aripiprazole [2]. Thereafter, other agents (asenapine, lurasidone, paliperidone, cariprazine, loxapine inhalation powder) have been studied and approved by FDA for either manic, mixed, or depressive phases of bipolar disorder or schizoaffective disorder. However, approval and current availability of individual agents vary across other countries.

### 4.5 Real World Versus Randomized Clinical Trials

As the acute response to treatment was beyond the scope of this survey, we focused on medication prescribed at discharge from the hospital. In fact, a hospital stay is one of the best opportunities to start (or reinforce) prophylaxis of mood disorders. Prophylaxis should be taken into account because mood disorders are often recurrent, affect the quality of life, and are associated with long-term increased mortality particularly due to suicide [5].

Unfortunately, controlled studies do not generally test long-term effectiveness because of the elevated costs of such trials. There are even discrepancies in the definitions of phases of mood disorders (*e.g.* relapses versus recurrences) and their therapy (*e.g.* continuation, maintenance, prophylaxis, *etc*.) [2]. It must also be underlined that the term “mood stabilizer” is not used by regulatory agencies.

Cipriani and colleagues [39] in their review of study designs of long-term studies for bipolar disorder underscored the problem of “enrichment design”: all randomized clinical trials selected patients who responded to acute treatment to increase the treatment effect observed in the long-term phase. By contrast, in the prescribing information sheets for all second-generation antipsychotics, the reported indication was 'maintenance treatment of bipolar disorder'. They concluded that “extrapolation of results from enrichment studies to the more general population of patients should be carried out cautiously because average treatment benefits are likely to be less in unselected patients” [39].

The results from our “real world” survey indicate that, even if the medication has been prescribed in compliance with the indications of regulatory agencies for acute episodes of mood disorders, the prescription attitude has differed significantly among hospital clinicians serving at the two psychiatric wards studied. Individual specialists make their own decision about the patients under their care.

Some choices might have been influenced by the current trend to limit hospital stays as much as possible (indeed, the mean stay averaged 8-10 days). This could be one of the reasons why benzodiazepines and more sedative first-generation or second-generation antipsychotics (*e.g.* promazine or quetiapine) were prescribed to most patients to control agitation, irrespective of diagnosis. Supposedly, sedative drugs were maintained at the time of discharge because the task of tapering or withdrawing them was left to the outpatient specialists once the agitation improved. With regard to benzodiazepines, it must be underscored that, in the 1980-1990s, prior to the advent of Divalproex and second-generation antipsychotics, adjunctive treatment with lorazepam or clonazepam was studied for acute mania in the US. For example, one double-blind study [40] concluded that lorazepam may offer an efficacious and safe alternative to haloperidol as an adjunctive treatment to lithium in the clinical management of the early phase of manic agitation in a subgroup of bipolar patients. However, as reviewed in a meta-analysis published in 2004, trial designs with benzodiazepines were considered heterogeneous and patient number was limited. The conclusion was that clonazepam is efficient and safe in the treatment of acute mania, but the results remain inconclusive for lorazepam [41].

Valproate may be prescribed more often than lithium thanks to its more rapid titration to achieve a quick therapeutic response [42]. In fact, for both humanitarian and economic reasons, interest in rapidly reducing symptoms of mania with pharmacologic loading strategies has increased [42].

Current biological treatment of acute agitation or aggression in the inpatient setting has recently been reviewed [42]. Among the pharmacologic agents studied in randomized controlled trials, second-generation antipsychotics have the best evidence to support efficacy [43].

### 4.6 Combined Medication

The extensive prescription of combined regimens is not unexpected. In fact, it has been shown that combined antipsychotic/mood stabilizer have efficacy advantages over antipsychotic or mood stabilizer monotherapy at least in acute mania, and should be considered as first-line therapy [44, 45]. However, the lower tolerability of combinations should also be taken into account.

In a systematic review, Fornaro and colleagues [46] reported that regardless of type or current mood episode polarity of bipolar disorder, prevalence rates up to 85% and 36% were found using the most permissive (two or more medications at once) and the most conservative (four or more) operative definitions for polypharmacy, respectively. Moreover, lithium prescription rates ranged from 13% to 33% in patients receiving polypharmacy according to conservative and permissive definitions, possibly suggesting a reduced need for augmentation of combination strategies for those cases with a favorable lifetime lithium response and/or long-lasting treatment as well as less likelihood of lithium response over the time most severe cases possibly exposed to a more complex polypharmacy overall.

### 4.7 Current Positions

With regard to individual agents, Bauer and Gitlin [47] commented that although first-generation antipsychotics are also effective, unquestionably, the second-generation antipsychotics now dominate the pharmacotherapy of acute mania. This reflects both their clear efficacy as shown in studies and meta-analysis, their ease of administration, relatively more benign side effect profiles (certainly compared to first-generation antipsychotics), and their rapidity of response compared to lithium.

However, the ideal mood stabilizer has to be effective against both mania and depression, without any risk of increasing the incidence of opposite episodes. Unfortunately, some antipsychotics can increase the risk of depression and antidepressants can trigger mania if used as monotherapy [48]. This problem is sometimes overlooked in the context of acute episodes, because the aim may be the short-term response.

### 4.8 Differences Between SPDC-1 and SPDC-2

The different lithium prescribing attitude on discharge deserves a particular discussion. In some patients, prior nonresponse or side effects may have influenced the confirmation or the discontinuation of lithium therapy: However, this cannot be ruled out from our data. It should be noted that abandoning lithium prophylaxis may be associated with an increase of suicide in high-risk patients, even in the event of an incomplete mood stabilization [49, 50].

No similar evidence is available regarding currently prescribed alternatives to lithium, including carbamazepine [51], valproate [30], and gabapentin [52]. Moreover, even a systematic review of data from randomized trials, that necessarily focused on shorter-term treatment periods, confirmed that lithium is effective in the prevention of suicide, deliberate self-harm, and death from all causes in patients with mood disorders, compared to placebo and other active treatments [53, 54].

Coryell reviewed early (1973-1976) and recent (2000-2003) lithium versus placebo trials, as well as those assigned to alternative mood stabilizers *versus* placebo and concluded that differing success rates in early and more recent maintenance trials strongly suggest a cohort effect in which the lithium responders have been relatively unavailable for being recruited into recent maintenance trials [6].

In an evaluation of real-world long-term outcome, lithium has recently been found as the most effective mood-stabilizer in a study of the comparative effectiveness of pharmacologic treatments in the prevention of psychiatric re-hospitalization in a Finnish nationwide cohort of 18,018 patients with bipolar disorder followed up for a mean of 7.2 years [55].

Similar conclusions were reported by a recent systematic review of non-randomized controlled observational studies [56]. Maintenance lithium monotherapy was associated with improved outcome compared with another mood stabilizer in monotherapy, including valproate, lamotrigine, olanzapine, quetiapine, unspecified anticonvulsants, carbamazepine/lamotrigine, unspecified atypical antipsychotics and unspecified antipsychotics. Lithium in combination with other mood stabilizers was found to be superior to lithium monotherapy in some analyses, but not in others [56].

The significant differences observed between SPDC-1 and SPDC-2 warrant some interpretation. Both wards are located in the same hospital and derive from the division of one single SPDC occurred in 2008. There was a tendency to keep the original staff of the old SPDC in SPDC-1, while the SPDC-2 personnel was new. Indeed, the mean age of the psychiatrists serving in 2016 differed between SPDC-1 and SPDC-2 (59.2 *vs* 50.8; *p* = 0 .017). We can speculate that the different professional experience has influenced both the diagnostic and the prescription attitude: perhaps SPDC-1 clinicians have been prone to diagnose patients with classical nosographic entities compared to their younger SPDC-2 colleagues who have often concluded for unspecified or for schizoaffective disorders. Similarly, the older SPDC-1 clinicians have preferred first-generation antipsychotics and the younger SPDC-2 clinicians have preferred second-generation antipsychotics. We can hypothesize that experienced clinicians keep their long-lasting habits and are less prone to novelty.

### 4.9 Tolerability

Another aspect that could be overlooked in the choice of therapy for acute episodes is the tolerability of psychotropic drugs. Cipriani and colleagues [15] underscored that evidence comparing drugs is poor, but clinicians and patients should consider different side effect profiles as an important issue to inform their choice. For example, their Cochrane review comparing haloperidol with other active treatments concluded that haloperidol is associated with less weight gain than olanzapine, but with a higher incidence of tremor and other movement disorders [15].

Efficacy advantages of co-treatment with antipsychotics and mood-stabilizers versus monotherapy should be balanced with its greater adverse event burden, including weight gain-related, extrapyramidal, tremor, sedation/somnolence, vomiting, and glucose/lipid-related adverse events [57]. With regard to the maintenance phase, lithium appears the least likely to cause substantial weight gain among mood stabilizers studied in randomized trials [6].

Aripiprazole can induce gastrointestinal disturbances (nausea and constipation) and movement disorders (in particular akathisia). Comparative trials with medicines other than haloperidol and lithium are few, so the precise position of aripiprazole in therapy remains unclear [58].

Aripiprazole appears to have a superior tolerability profile when used as maintenance treatment. Side effects like a headache, insomnia, and extrapyramidal side effects, such as tremor and akathisia may be treatment limiting in some cases [59]. Aripiprazole was also associated with higher levels of high-density lipoprotein, but no difference in extrapyramidal symptoms in the maintenance phase versus placebo or in comparison with other medication such as haloperidol or lithium [60].

Besides tolerability of the acute and the immediately following phase, that have been addressed by controlled trials, the choice of clinicians should consider potential adverse events associated with longer-term treatment in the real world. For example, the aforementioned recent Finnish nationwide cohort study examined hospitalizations owing to physical illness (besides psychiatric re-hospitalizations) for 7.2 years [55]. In the comparison between the three most frequently used medication, lithium was associated with the lowest incidence of subsequent hospitalization owing to physical illness, followed by valproate and quetiapine [55].

It must be underscored that the 7.2-year follow-up could not reveal any adverse event associated with longer-term exposure, including the decline in renal function observed in patients treated with lithium for decades [61, 62].

Acute treatment with valproate has commonly been associated with nausea, vomiting, diarrhea, sedation, and tremor. Long-term treatment can also induce alopecia and polycystic ovarian disease. Hepatic and pancreatic toxicity have been reported, as well as an unusual encephalopathy associated with hyperammonemia. Dealberto reviewed fourteen cases of encephalopathy in the psychiatric setting published between 1995 and 2005: Ammonia concentrations varied across the reported cases from normal to ten-fold the upper limit of the normal range [63]. Raja and Azzoni found a correlation between valproate concentration and ammonemia in patients hospitalized in a psychiatric ward [64]. We confirmed that valproate can increase ammonemia [65]. Median concentrations were significantly higher in a group of 42 outpatients (20 µmol/L) treated with valproate for a median of 48 months compared with the group of 44 outpatients treated with other mood stabilizers (12.5 µmol/L). However, clinically relevant concentrations were rare [65].

## 5 SPECULATION

Sometimes, prescription attitudes in the real world are not completely evidence-based. Some differences can be attributed to the aforementioned variations across national regulatory agencies. One of the potential factors may be the increasing influence of American psychiatric literature worldwide.

In the 1980s, before the advent of second-generation antipsychotics and divalproex, ICD-9 was the official diagnostic system in use but DSM-III have also introduced in Italy along with its new concept of affective disorders (including those with mood-congruent and mood-incongruent psychotic features). Acute mania was generally treated with first-generation antipsychotics and/or benzodiazepines, based on severity. Lithium or carbamazepine were also frequently prescribed.

DSM-IV and ICD-10 were introduced in the 1990s. The first controlled Divalproex study for mania was published in 1994 [66], and the use of second-generation antipsychotics began to extend from schizophrenia to mood disorders [67].

Meanwhile, the efficacy of lithium was questioned particularly by Joanna Moncrieff in a series of papers published in very influential British and American psychiatric journals. She strongly challenged the validity of the pioneering trials of lithium prophylaxis in bipolar disorder [68-71]. She rekindled the “therapeutic myth”, suggesting that these trials “produced spurious results owing to flawed methods”, and concluded that “a close reexamination of these studies” was due. In the US, a dramatic change in prescription patterns followed the 1994’s Divalproex study at the expense of lithium, as confirmed by several surveys [28-30].

Thereafter, numerous studies and meta-analyses clearly demonstrated the efficacy/effectiveness of lithium, which has been used as a comparator in modernly designed trials.

Moreover, with regard to longer term outcome, it must be underscored that in the only random assignment study, valproate was generally less effective than lithium as maintenance treatment in bipolar disorder [72, 73], and the European Medicine Agency has withdrawn valproate’s indication as a maintenance treatment.

It must also be underlined that in the 2000-2010s, as commented by Bauer and Gitlin [47], aggressive pharmaceutical firm marketing of these agents [second-generation antipsychotics] has certainly added to their popularity.

## CONCLUSION

Although the treatment of acute mood disorders may be in line with randomized clinical trials and treatment guidelines, rates of prescription of individual psychotropic agents may differ significantly, as in the two psychiatric wards in this study. It must be underscored that different prescription attitudes can have relevant consequences on the long-term outcome of patients in terms of effectiveness, tolerability and mortality, as suggested by observational studies carried out in the “real world”.

## Figures and Tables

**Fig. (2):**
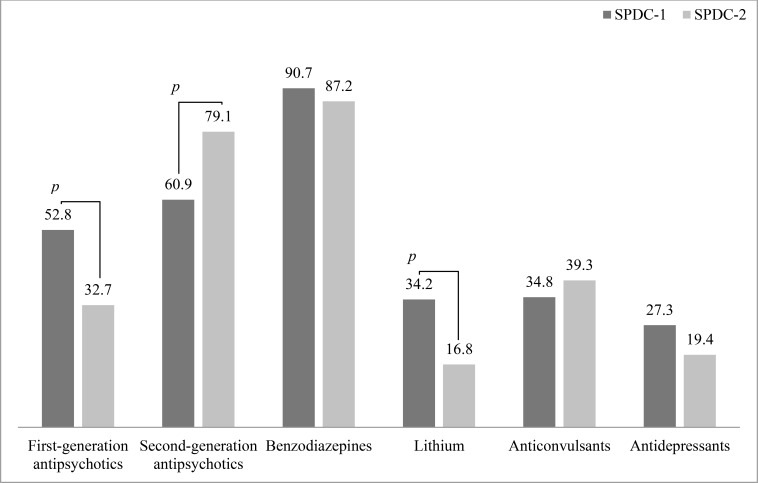
Rates (%) of medication prescribed at discharge from the two psychiatric wards SPDC-1 (n. of patients = 161) and SPDC-2 (n. of patients = 196).

**Table 1:** Demographic and clinical characteristics of the 357 patients hospitalized in the two psychiatric wards SPDC-1 and SPDC-2.

–	**SPDC-1 (n = 161)**	**SPDC-2 (n = 196)**
**Female, n (%)**	93 (57.8)	115 (58.7)
**Male, n (%)**	68 (42.2)	81 (41.3)
**Current age, mean (± SD)**	46 (±13.7)	47.4 (±13.1)
**Days of hospitalization (mean)**	10.5 (±7.8) *	8.1 (±8.1)*
**Age at onset, mean (± SD)**	32 (±15)	30 (±12.4)
**Hospitalized for the first time, n (%)**	59 (36.6)	80 (40.8)
**History of suicide attempts, n (%)**	52 (32.3)	50 (26.5)
**History of alcohol abuse, n (%)**	18 (11.2)	29 (14.8)
**History of drug abuse, n (%)**	23 (14.3)	28 (14.3)

*p = 0.0045

**Table 2:** Medication prescribed at discharge from the hospital.

Drugs n, (%)		SPDC 1 (n =161)	SPDC 2 (n = 196)	*p*
First-generation antipsychotics				
	haloperidol	47 (29.2)	33 (16.8)	**0.0072**
	promazine	29 (18.0)	25 (12.8)	0.1834
	chlorpromazine	7 (4.3)	3 (1.5)	0.1952
	others	2 (1.2)	1 (0.5)	0.5908
Second-generation antipsychotics				
	quetiapine	40 (24.8)	55 (28.1)	0.5479
	aripiprazole	18 (11.2)	38 (19.4)	**0.0404**
	olanzapine	9 (5.6)	26 (13.3)	**0.0192**
	asenapine	11 (6.8)	6 (3.1)	0.1330
	clozapine	9 (5.6)	10 (5.1)	0.4322
	risperidone	9 (5.6)	13 (6.6)	0.8258
	others	6 (3.7)	7 (3.6)	1.0000
Anticonvulsants				
	valproate	40 (24.8)	53 (27.0)	0.7164
	carbamazepine	12 (7.5)	19 (9.7)	0.5717
	lamotrigine	5 (3.1)	5 (2.6)	0.7591
	others	7 (4.3)	18 (9.2)	0.0953
Lithium		55 (34.2)	33 (16.8)	**0.0002**

Differences in proportions were calculated with Fisher’s exact test. *P* values < 0.05 are displayed in bold.

## LIST OF ABBREVIATIONS

SPDC: = Servizio Psichiatrico di Diagnosi e Cura (Psychiatric Diagnosis and Care Service).
FDA: = Food and Drug Administration
